# Exploitation of dihydroorotate dehydrogenase (DHODH) and p53 activation as therapeutic targets: A case study in polypharmacology

**DOI:** 10.1074/jbc.RA119.012056

**Published:** 2021-01-13

**Authors:** Marcus J. G.W. Ladds, Gergana Popova, Andrés Pastor-Fernández, Srinivasaraghavan Kannan, Ingeborg M.M. van Leeuwen, Maria Håkansson, Björn Walse, Fredrik Tholander, Ravi Bhatia, Chandra S. Verma, David P. Lane, Sonia Laín

**Affiliations:** 1 Department of Microbiology, Tumor and Cell Biology, Karolinska Institutet, Stockholm, Sweden; 2 SciLifeLab, Department of Microbiology, Tumor and Cell Biology, Karolinska Institutet, Stockholm, Sweden; 3 Bioinformatics Institute (BII), A*STAR, Singapore; 4 SARomics Biostructures AB, Lund, Sweden; 5 Department of Medical Biochemistry and Biophysics, Karolinska Institutet, Stockholm, Sweden; 6 Division of Hematology and Oncology, O'Neal Comprehensive Cancer Center, University of Alabama, Birmingham, Alabama, USA; 7 Department of Biological Sciences, National University of Singapore, Singapore; 8 School of Biological Sciences, Nanyang Technological University, Singapore

**Keywords:** molecular pharmacology, p53, mitochondria, tumor cell biology, cell death, molecular modeling, nucleoside/nucleotide biosynthesis, nucleoside/nucleotide transport

## Abstract

The tenovins are a frequently studied class of compounds capable of inhibiting sirtuin activity, which is thought to result in increased acetylation and protection of the tumor suppressor p53 from degradation. However, as we and other laboratories have shown previously, certain tenovins are also capable of inhibiting autophagic flux, demonstrating the ability of these compounds to engage with more than one target. In this study, we present two additional mechanisms by which tenovins are able to activate p53 and kill tumor cells in culture. These mechanisms are the inhibition of a key enzyme of the *de novo* pyrimidine synthesis pathway, dihydroorotate dehydrogenase (DHODH), and the blockage of uridine transport into cells. These findings hold a 3-fold significance: first, we demonstrate that tenovins, and perhaps other compounds that activate p53, may activate p53 by more than one mechanism; second, that work previously conducted with certain tenovins as SirT1 inhibitors should additionally be viewed through the lens of DHODH inhibition as this is a major contributor to the mechanism of action of the most widely used tenovins; and finally, that small changes in the structure of a small molecule can lead to a dramatic change in the target profile of the molecule even when the phenotypic readout remains static.

Polypharmacology, the ability of a pharmacological agent to bind to and alter the function of multiple targets, is thought of as a double-edged sword in drug development programs (1). Whereas multiple targeting may be a desired approach in complex diseases, such as cancer, where inhibition of multiple pathways is more efficacious in promoting tumor cell death and for overcoming resistance to therapy, the risk of increasing off-target toxicities is a legitimate concern. Therefore, the use of “fingerprinting” of compounds to mitigate adverse drug reactions using safety panels has become more commonplace during the drug development process (2, 3, 4). Nevertheless, many currently approved drugs do indeed hit multiple targets, and this in turn can contribute to their efficacy (5, 6, 7). One school of thought actually promotes the idea of hitting multiple desirable targets with one agent, rather than using multiple agents to hit single targets as a means to reduce unwanted dose-limiting toxicity that can occur upon the combination of multiple pharmacological agents (8, 9, 10).

Tenovin 6 was originally discovered as an inhibitor of SirT1 and SirT2 (11). The ability of tenovins to block autophagic flux has been recently found to be a property of the tertiary aliphatic amine that was added to the structure of tenovin 1 to increase aqueous solubility (12, 13, 14). Through structure-activity relationship studies using our previously published tenovin analogues (14, 15), we uncover the ability of certain tenovins to inhibit two novel pathways: the *de novo* pyrimidine synthesis pathway by inhibiting DHODH, and also nucleoside transport. The blockage of DHODH by another chemical class adds to our previous findings that DHODH is a frequently hit target of small molecules (16). Taken together, this study suggests that polypharmacology may be currently exploited unknowingly, to achieve tumor cell elimination, and that more compounds, particularly those that are acidotropic with hydrophobic regions, may also interact with DHODH.

## Results

### Tenovins are capable of inhibiting DHODH activity

**Figure 1:**
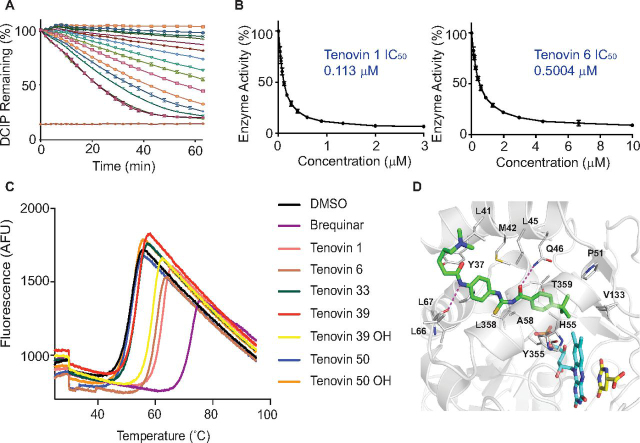
**Tenovins can inhibit DHODH.***A*, a dose titration of tenovin 1 in the enzymatic assay. Curves show the color reduction in DCIP over time for illustrative purposes. *B*, values obtained using a kinetic DHODH enzyme assay. Values correspond to the average of three independent repeats ± S.D. with three technical repeats each. *C*, thermal denaturation curve of DHODH incubated with various tenovins (200 μm) and brequinar (200 μm) as a positive control. *D*, co-crystal structure of tenovin 6 in complex with the enzyme DHODH. DHODH is shown as a *cartoon* (*gray color*), and the cofactor FMN (*cyan carbons*), the substrate DHO (*yellow carbons*), and bound tenovin 6 (*green carbon*) are shown as *thick lines*. The tenovin 6 binding pocket residues are denoted as *thin lines*, and the hydrogen bonds between DHODH and tenovin 6 are shown as *dashed lines* (*magenta*). Details of the crystal structure can be found in Table S1.

We recently described how a wide variety of small molecules with a highly diverse array of unrelated structures activate p53 and do so by inhibiting DHODH (16). This encouraged us to test whether the tenovins could also inhibit this enzyme. We used an enzyme activity assay to assess the effect of tenovins on DHODH enzymatic activity (Fig. 1*A*). This assay revealed that a number of tenovin analogues are capable of inhibiting DHODH (IC_50_ shown in Fig. 1*B* and Fig. S2). In addition, we conducted a thermal shift assay utilizing the intrinsic fluorescence of the flavin FMN cofactor that is liberated upon thermal denaturation and unfolding of the protein (Fig. 1*C*). This assay displayed a generally good agreement with the *in vitro* enzymatic activity assay, implying that target engagement leads to the inhibition of enzymatic activity. Despite this, the thermal shift assay suggested that tenovin 39OH was capable of stabilizing DHODH to a similar extent as the other tenovins capable of inhibiting DHODH enzymatic activity. This is surprising, as tenovin 39OH only demonstrated a mild inhibitory effect in the enzymatic assay with an IC_50_ of around 5.8 μm (Fig. S2), an IC_50_ that is ∼10 times that of tenovin 6 and 50 times that of tenovin 1 (see “Discussion” for further elaboration on this topic). We finally obtained a crystal structure of tenovin 6 co-crystallized with DHODH (Fig. 1*D*). This crystal structure demonstrated that the tenovins bind in the same pocket as our previously published compound, HZ05 (16) as well as brequinar and teriflunomide (17).

### Modeling the interaction with DHODH and SirT1

We previously conducted a target confirmation study with the tenovins (see Table 1 for structures) by examining their ability to interact with SirT1 in cells using a cellular thermal shift assay (CETSA) to ensure that the compound series interacted with its purported target (14). In the present study, we have used molecular modeling to examine the energetics of interactions of these molecules in their interaction with SirT1 (Fig. S1, A and B). This modeling study and its correlation with the results seen in both the enzymatic assay and thermal shift data (11, 14, 18) served as a proof of principle for modeling the interactions of the tenovins with other targets.

**Table 1:** Structures of tenovins in this study

Compound	R	R_1_

Tenovin 1	S	H
Tenovin 6	S	(CH_2_)_4_NMe_2_
Tenovin 33	O	(CH_2_)_4_NMe_2_

Tenovin 39	S	NMe_2_
Tenovin 39OH	S	OH
Tenovin 50	O	NMe_2_
Tenovin 50OH	O	OH

**Figure 2:**
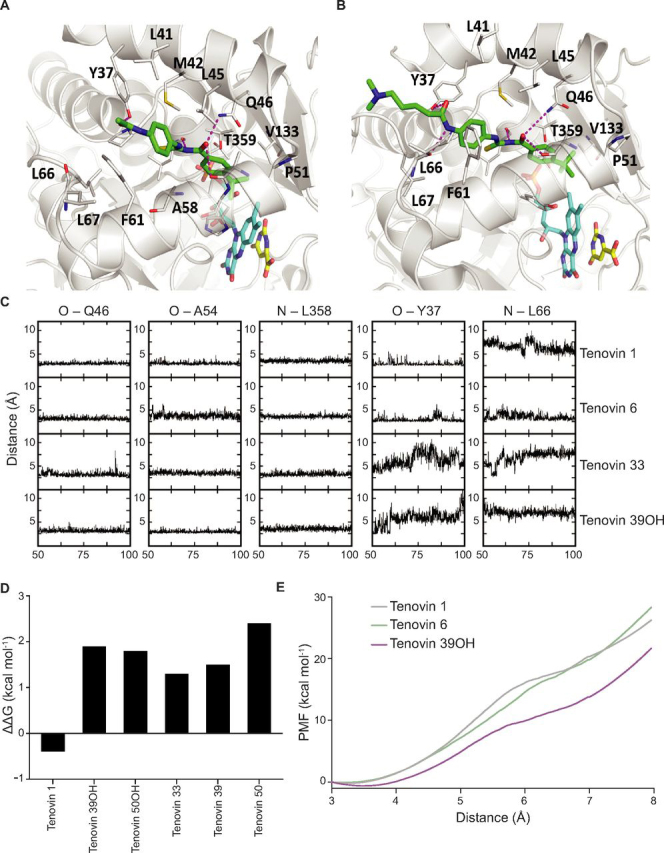
**Modeling the interaction between the tenovins and DHODH.** Shown is the predicted binding mode of tenovin 1 (*A*) and tenovin 6 (*B*) in complex with DHODH. The enzyme DHODH is shown as a *cartoon* (*gray color*), and the cofactor FMN (*cyan carbon*), the substrate DHO (*yellow carbon*), and bound tenovins (*green carbon*) are shown as *thick lines*. The tenovin-binding pocket residues are shown as *thin lines*, and the hydrogen bonds between DHODH and tenovins 1 and 6 are shown as *dashed lines* (*magenta*). *C*, time evolution of the DHODH–tenovin interactions. Distances between the atom pairs are calculated for the conformations sampled during the second half of the simulations. *D*, computed free energies differences between the binding of tenovins to DHODH in the membrane relative to the binding of tenovin 6 to DHODH in the membrane (negative value implies tighter binding of a particular tenovin relative to tenovin 6); the FEP/MBAR method was used. *E*, the free energies required to pull tenovin 1, 6, or 39OH from the binding pocket on DHODH. Positions of the three tenovins relative to DHODH are shown in Fig. S1C.

Based on the crystal structure of tenovin 6 co-crystallized with DHODH, we decided to model the potential interactions of the tenovins with DHODH to examine the energetic favorability of the interaction. Our structural models were constructed using the apo-structure of DHODH and refined using extensive molecular dynamics (MD) simulations. The models suggest that tenovin 1 possesses the same predicted interaction at Gln-46 as tenovin 6 (Fig. 2, *A* and *B*) and that the reversed amide present in tenovin 39, tenovin 39OH, tenovin 50, and tenovin 50OH is not favored.

The stability of the interactions of the tenovins with DHODH was determined by examining the temporal evolution of key interatomic distances between atoms of tenovin 1, 6, 33, and 39OH and atoms of DHODH during the MD simulations (Fig. 2*C*). The substitution of a urea for the thiourea in tenovin 33 led to a less stable interaction at Tyr-37 as compared with both tenovin 1 and 6. Additionally, the analysis also suggested that the reversed amide of tenovin 39OH was disfavored, with poorer interactions at Tyr-37 and Leu-66 resulting in destabilized energies of interactions compared with tenovin 1 (Fig. 2*D*). Adding further weight to these simulations, the free energy costs associated with pulling the ligands out of the binding pocket of DHODH, a surrogate for *k*_off_, were carried out for tenovin 1, 6, and 39OH (Fig. 2*E*). It is clear that tenovin 39OH requires less free energy to unbind from DHODH, suggesting its markedly less favorable energetics of binding compared with that of tenovins 1 and 6.

### The tenovins activate p53 and kill cancer cells through different mechanisms

**Figure 3:**
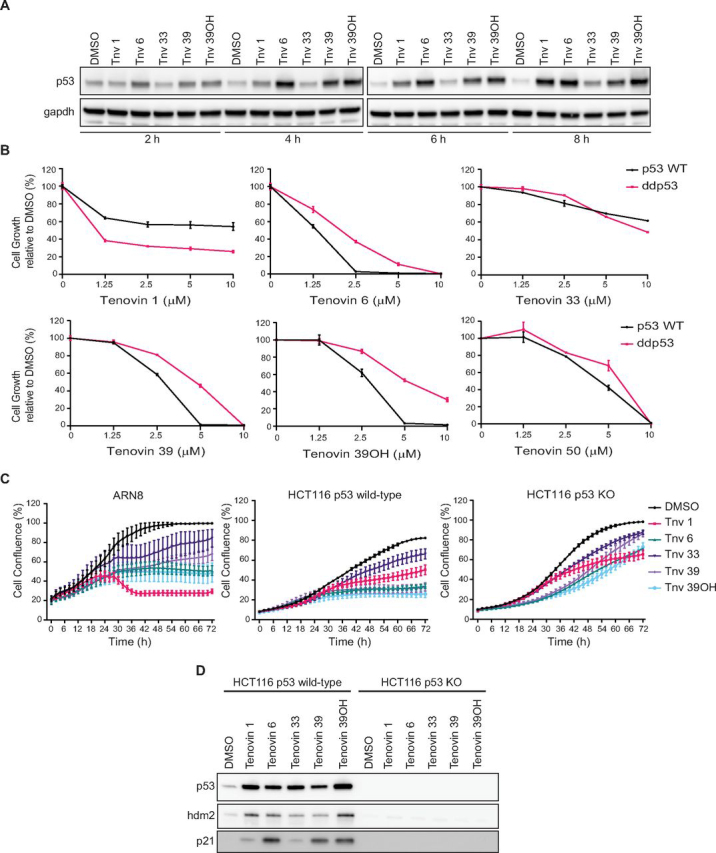
**Analysis of p53 dependence in the mechanism of action of the tenovins.***A*, Western blotting of p53 levels in ARN8 cells upon treatment with a 5 μm concentration of the indicated tenovins for either 2, 4, 6, or 8 h. *B*, SKNSH cells with normal WT p53 or stably expressing ddp53 were treated with a dose titration of the indicated tenovins for 72 h. Results are a single representative experiment with three technical replicates ± S.D. A total of three biological replicates were conducted. *C*, ARN8, HCT116 p53 WT, or p53 KO cells were treated with a fixed 5 μm dose of each tenovin and monitored for the confluence of the culture over 72 h by IncuCyte. Results are a single representative experiment with three technical replicates ± S.D. A total of three biological replicates were conducted. *D*, HCT116 p53 WT or p53 KO cells were treated with a 5 μm concentration of the indicated tenovins for 24 h prior to blotting for p53 and p53 targets. The total protein loading control for these blots is shown in Fig. S4.

We have, in previous studies, examined the ability of tenovins to enhance p53 transcriptional activity in a cell-based reporter assay (β-Galactosidase (CPRG) assay) as well as affect cell viability (11, 18). Following the discovery that certain tenovins inhibit DHODH, we examined the contribution of DHODH inhibition to their mechanism of action. As it has been previously established that tenovin 1 and tenovin 6 are capable of activating p53 (11), we decided to examine the increase in p53 levels by each tenovin over time (Fig. 3*A*). We observed that tenovins 6, 39, and 39OH were quickest at increasing p53 protein levels and did so after only 2 h of treatment. In contrast, tenovins 1 and 33 were slower at increasing levels of p53 in cells, with tenovin 1 showing a slight increase at 4 h and tenovin 33 showing a slight increase at 8 h. Following this, we decided to check the contribution of p53 to the ability of the tenovins to reduce tumor cell growth. We used SKNSH cells that express WT p53 or an isogenic cell line stably expressing ddp53 (also known as DNp53)—a dominant negative truncated form of p53—as described previously (19). As can be seen, tenovins 6, 39, and 39OH had the ability of tenovin 6, 39, 39OH to kill SKNSH cells when ddp53 was expressed whereas the effectiveness of tenovin 50 was not markedly changed (Fig. 3*B*). In contrast, tenovin 1 was more able to reduce the growth of cells expressing ddp53, implying that other factors in these cells predisposed them to the effects of tenovin 1 and that the mechanism by which tenovin 1 kills cells is not dependent on p53. Indeed, it has been shown that treatment with a DHODH inhibitor can cause differentiation and cell death in p53-deficient acute myeloid leukemia cells (20). However, it must be noted that we previously demonstrated that tenovin 1 causes death as well as accumulation of cells in the G_1_ phase of the cell cycle in SKNSH cultures (11). In contrast, in cultures of SKNSH cells expressing ddp53, this tenovin promotes the accumulation of cells in S phase (11). We conclude that the type of effect of tenovin 1 on the cell cycle and cell viability depends on the activity of p53 even if the overall effect on cell growth does not. Following these results, we also went on to test the dynamics of tumor cell growth arrest in HCT116 p53 WT and p53 KO cells (21). As can be seen in Fig. 3*C*, and in agreement with our results in SKNSH cells, tenovins 6, 39, and 39OH had their effects on cell growth ablated in the p53 KO cells. In contrast, the activity of tenovin 1 was relatively unaffected. To confirm the absence of p53 signaling in these cells, we conducted Western blotting for p53 protein, p21, and HDM2 and confirmed that those cells capable of bringing up p53 protein were also able to induce expression of HDM2 and p21 in the HCT116 p53 WT, but not the p53 KO cells (Fig. 3*D*).

**Figure 4:**
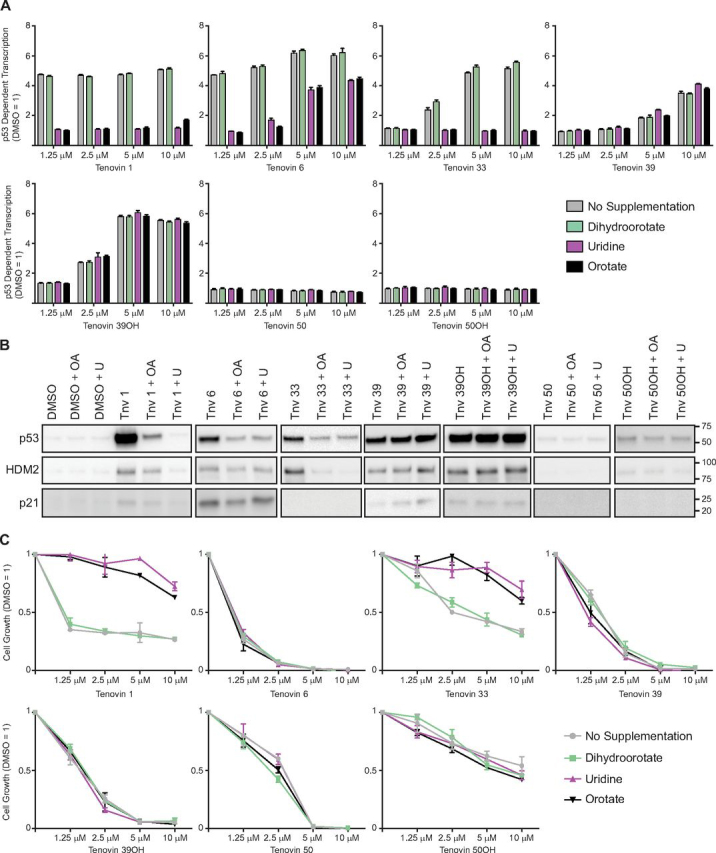
**Phenotypic analysis of tenovins upon supplementation with nucleotides.***A*, p53 transcriptional activity assay (CPRG) in ARN8 cells treated with tenovins for 18 h supplemented with 100 μm uridine, 1 mm DHO, or 1 mm OA. Results are a single representative experiment with three technical replicates ± S.D. A total of three biological replicates were conducted. *B*, Western blotting of p53 and p53 targets in ARN8 cells treated for 24 h with a 5 μm concentration of the indicated tenovins supplemented with 100 μm uridine, 1 mm DHO, or 1 mm OA. The total protein loading for these blots is shown in Fig. S4. *C*, SRB assay in ARN8 cells treated with the indicated tenovins for 72 h supplemented with 100 μm uridine, 1 mm DHO, or 1 mm OA. Results are a single representative experiment with three technical replicates ± S.D. A total of three biological replicates were conducted.

As a follow-up to our studies on the role of p53 in the activity of the tenovins, we decided to examine the contribution of DHODH inhibition to the ability of the tenovins to induce p53. We therefore supplemented the cell culture media with either excess uridine, the product (orotate), or substrate (dihydroorotate) of the DHODH reaction to see whether or not the induction of p53 transcriptional activity by the tenovins was affected. As can be seen in Fig. 4*A*, the induction of p53 transcriptional activity by tenovins 1 and 33 was completely ablated by the addition of orotate (OA) or uridine, whereas they were capable of activating p53 in the presence of dihydroorotate (DHO). Tenovin 6, the second-best inhibitor of DHODH in this panel of compounds, had its p53 transcriptional activity partially ablated upon the addition of uridine or OA, but not DHO. The activities of tenovins 39 and 39OH were unaffected upon supplementation with DHO, OA, or uridine. Tenovin 50 and 50OH, as reported previously, were unable to activate p53 and were unaffected by the addition of DHO, OA, or uridine. To add to these assays, we carried out Western blotting for p53 protein and two of its downstream effectors, hdm2 and p21 (Fig. 4*B*). In agreement with the CPRG assay, the addition of OA and uridine caused an ablation of p53 downstream signaling in tenovin 1–, tenovin 6–, and tenovin 33–treated cells as well as reducing p53 protein levels, whereas p53 protein and signaling in tenovin 39– and tenovin 39OH–treated cells were largely unaffected. Tenovin 50 and tenovin 50OH were unable to bring up levels of p53 or its downstream targets.

**Figure 5:**
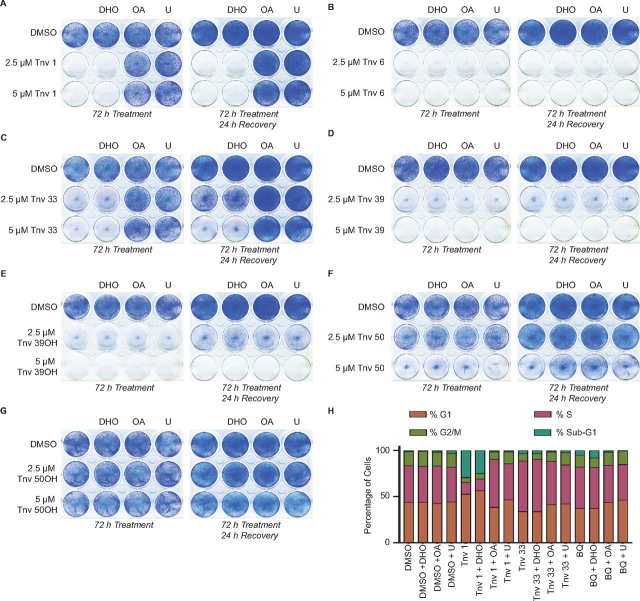
**Cell viability and cell cycle effects of tenovins upon supplementation with nucleotides.***A–G*, ARN8 cells treated for 72 h with compound or treated for 72 h followed by compound wash-out and recovery in fresh medium for 24 h. *H*, ARN8 cells treated for 48 h with the indicated compounds at 10 μm (tenovins 1 and 33) or 200 nm (brequinar) with or without supplementation with 100 μm uridine, 1 mm DHO, or 1 mm OA.

Following the investigation into the role of DHODH inhibition on p53, we then conducted cell viability assays to examine the effect of supplementation on melanoma cell growth using a sulforhodamine B viability (SRB) assay (Figs. 4*C* and 5 and Fig. S3). The effect of tenovins 1 and 33 on tumor cell growth was reduced upon supplementation with uridine or OA, which mirrors the effect of supplementation with OA or uridine on their ability to induce p53-dependent transcription. Supplementation elicited no rescue of cell viability in the case of tenovin 6 treatment despite the partial ablation of p53 transcriptional activation, suggesting that DHODH inhibition is not the main mechanism by which tenovin 6 kills melanoma cells. The activity of tenovins 39 and 39OH was unaffected by supplementation. The SRB assay results were further reinforced by clonogenic studies that recapitulated the phenotype seen in the SRB assays (Fig. 5, *A–G*). To pursue these studies further, we investigated the effect of supplementation with uridine, DHO, or OA on the cell cycle in cells treated with tenovins whose effects were recovered by OA or uridine in the viability assays. We therefore conducted propidium iodide flow cytometry analysis. Much as we observed previously (16), DHODH inhibition resulted in an increase in the percentage of cells in S-phase, as noted with tenovin 33 and brequinar in particular, and an increase in cell death with tenovin 1. We also observed a correlation between DHODH inhibition and the restoration of the normal cell cycle distribution upon supplementation with OA or uridine. As seen in Fig. 5*H*, tenovins 1 and 33 had their effect on the cell cycle largely prevented, and the cells showed a similar phase distribution to that seen in DMSO treated cells upon the addition of uridine or OA, but not upon supplementation with DHO.

### Uridine uptake is perturbed by tenovins

Cells possess both a *de novo* and salvage pathway for synthesis of pyrimidine nucleotides. As it had been established previously that a plethora of compounds are capable of inhibiting nucleoside transport into cells (22), we investigated whether tenovins could also block exogenous uridine uptake. This is of particular relevance when considering DHODH as a therapeutic target because uridine is present in the blood and therefore may feed into the pyrimidine salvage pathway, counteracting the effect of DHODH inhibition (23). We have seen previously that uridine, at concentrations that are reported to be present in blood, does not fully recover the effect of DHODH inhibitors, but it does partially dampen their efficacy (16).

**Figure 6:**
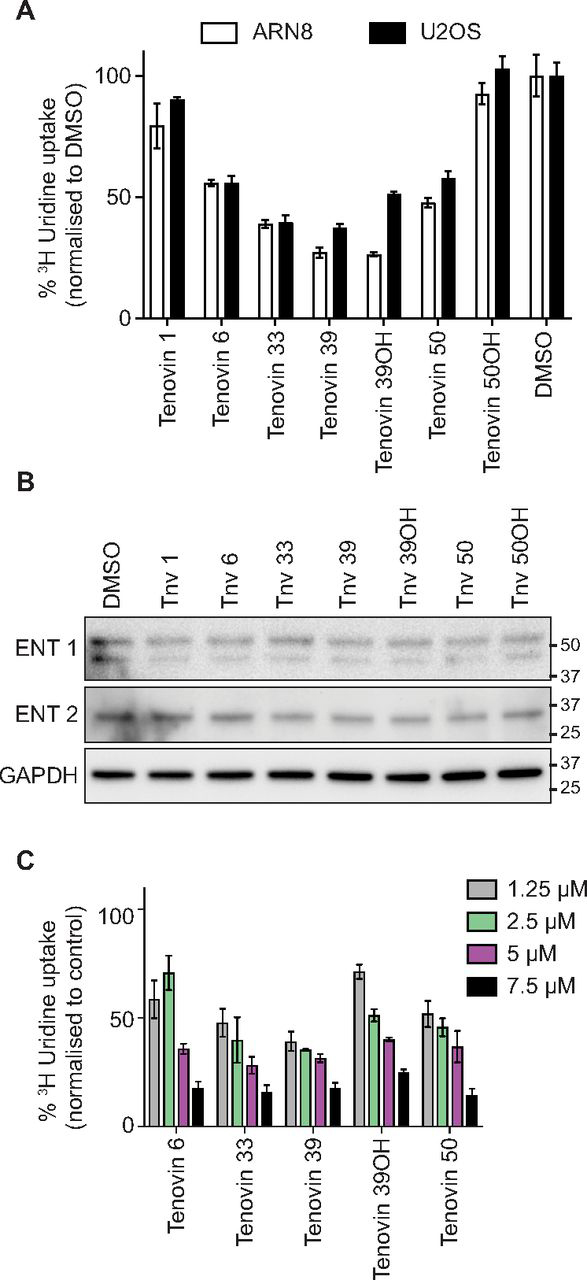
**Inhibition of uridine uptake by tenovins.***A*, ARN8 or U2OS cells treated for 15 min with a 5 μm concentration of the indicated compounds, with uptake of ^3^H uridine measured by a scintillation counter. All data are normalized to protein levels prior to normalization to DMSO control (DMSO = 100). *Error bars*, S.D. of three technical replicates. *B*, Western blotting of U2OS cells treated for 24 h with a 5 μm concentration of the indicated compounds. *C*, U2OS cells treated for 24 h with the indicated compounds, with uptake of [^3^H]uridine measured by a scintillation counter. All data are normalized to protein levels for each individual compound as a proxy for cell number and then normalized to the DMSO control (DMSO = 100). *Error bars*, S.D. of three technical replicates.

We observed that, following 15 min of incubation with either ARN8 or U2OS cells, certain tenovins were capable of inhibiting the uptake of uridine from the medium. Tenovins 39 and 50 were able to inhibit uridine uptake into cells while not inhibiting DHODH (Fig. 6*A*). Tenovin 50OH was unable to inhibit DHODH or uridine uptake. Tenovin 6 and 33 were able to inhibit uridine uptake and also inhibit DHODH. Tenovin 1 was unable to alter uridine uptake, although it was the most potent DHODH inhibitor from the tenovin series tested. Using the tenovins that were able to inhibit uridine uptake, we investigated whether the effect was dose-dependent and persistent by testing four different concentrations of each tenovin capable of blocking uridine uptake after 15 min and incubating them with the cells for 24 h. We used U2OS cells, cells that are more resistant to cell killing by tenovins, to assess their effect on uridine uptake as ARN8 cells started to die after 24 h, making quantitation of uptake and relative expression of ENTs more difficult. This was considered to be a viable proxy, as both the U2OS and ARN8 cells showed the same response with regard to uridine uptake inhibition after 15 min of tenovin incubation (Fig. 6*A*).

Prior to conducting uridine uptake studies, we assessed whether any tenovin led to a change in the expression of the main equilibrative transporters for nucleotides, ENT1 and ENT2. We determined ENT1/2 levels in U2OS cells treated with each of the tenovins for 24 h and saw no appreciable change in ENT1 or ENT2 levels (Fig. 6*B*). Furthermore, all tenovins that were capable of inhibiting uridine uptake after 15 min were also able to inhibit uptake after 24 h in a dose-dependent manner, confirming that the effect on uridine uptake was not a transient event.

## Discussion

As the field of chemical biology field becomes markedly more reductionist in its approach to targeting diseases with highly specific compounds that interact with their target in the subnanomolar range, we highlight here a series of compounds that hit multiple pathways. Exquisitely specific therapies against one or few targets are often highly effective but are rarely given as single agents and are usually administered as part of a combination regime. Therefore, the idea of a “magic bullet” compound that hits multiple pathways at once to eliminate tumor cells becomes more attractive. By administering one or few compounds, one increases the possibility of reducing the complex pharmacology of multiple dosing, thus lowering the risk of adverse events (24, 25).

We have uncovered DHODH as a new target for tenovins 1, 6, 33, and 39OH, both using biochemical assays for enzyme activity and in phenotypic structure-activity relationship studies. This finding holds wide-ranging implications for any research utilizing these tenovins. Equally, the inhibition of uridine uptake, most likely through the inhibition of equilibrative nucleoside transporters, demonstrated that small changes to the molecule lead to switching of the targeting profile. This is exemplified by comparing tenovin 6, a potent DHODH inhibitor and blocker of uridine uptake, with tenovin 39, which is incapable of DHODH inhibition but diminishes uridine uptake, or with tenovin 1, which is a potent DHODH inhibitor and does not block uridine uptake. These changes in targeting profile occur despite the structural similarity between these three compounds.

As summarized in Table 2, we can see that only tenovin 6 is capable of inhibiting all targets examined in these structure-activity relationship studies. In contrast, tenovin 1 has been shown to inhibit DHODH, whereas its effects on SirT1 have been difficult to ascertain by using an *in vitro* enzymatic activity assay due to solubility issues at high concentrations (11, 18). Importantly, there have been several studies that have utilized tenovin 1 recently in a variety of contexts that assume that its inhibition of SirT1 is its primary mechanism of action (26, 27, 28, 29). Tenovin 1 is a very potent inhibitor of DHODH, with an IC_50_ of ∼113 nm. These new findings may put other studies into a more understandable context when considering the ability of tenovin 1 to inhibit DHODH and the role this plays in its mechanism of action.

**Table 2:** Summary of tenovin-targeting profiles

Compound	Blockage of autophagic flux^a^	p53 transcriptional activity induction	DHODH inhibition (enzyme assay)	DHODH inhibition (phenotypic identification^b^)	Uridine uptake inhibition	SirT1 target engagement or biochemical inhibition^c^
			μ*m*			
Tenovin 1	−	+	0.113	+	–	NT
Tenovin 6	+	+	0.5004	+	+	+
Tenovin 33	+	+	>20	+	+	+
Tenovin 39	+	+	>20	−	+	+
Tenovin 39OH	−	+	5.8	−	+	+
Tenovin 50	+	−	>20	−	+	+
Tenovin 50OH	−	−	>20	−	−	NT


a Based on data presented in Ref. 12.

b Based on recovery of p53 transcriptional activity induction and recovery upon the addition of uridine or orotate.

c Based on cellular thermal shift data from Ref. 12 and data presented in Ref. 16. Tenovins 1 and 50OH were not tested (NT) in the target engagement study (CETSA) due to solubility issues at the concentrations required for this test (12).

Tenovin 39 and its closely related analog, tenovin 39OH, demonstrate markedly different abilities to inhibit DHODH. Despite the aliphatic hydroxyl group not binding to DHODH directly, tenovin 39OH is capable of inhibiting DHODH enzyme activity and stabilizing DHODH in a thermal shift assay. In contrast, as seen in Table 1, tenovin 39, which possesses a terminal tertiary amine at the end of the aliphatic chain instead of the hydroxyl group, is incapable of inhibiting DHODH. This study demonstrates the complex mechanisms that govern the manner in which molecules differ in their abilities to target a particular protein even when the differences between molecules are localized in their seemingly noninteracting regions. Other tenovins in possession of tertiary amines, such as tenovin 6 and tenovin 33, are capable of inhibiting DHODH, and thus the presence of the tertiary amine does not appear to be intrinsically disfavored. It is also highly important to note that there is no phenotypic readout for the inhibition of DHODH upon tenovin 39OH treatment of cells, suggesting that the high IC_50_ observed in the enzyme activity assay (5.8 μm) may mean that insufficient concentration of tenovin 39OH is reached in cells to inhibit the enzyme. Our ligand-pulling simulations also strongly suggest that the energetics of tenovin 39OH entering the quinone tunnel of DHODH are less favorable than the interactions seen with tenovins 1 and 6. This may serve to provide an explanation for the evidence that DHODH inhibition does not contribute to the mechanism of action of tenovin 39OH.

In contrast to tenovin 39OH, tenovin 33 is an enigmatic compound for a different reason. In the biochemical assays, tenovin 33 was incapable of inhibiting DHODH enzyme activity; nor was it able to stabilize DHODH in a thermal shift assay. Yet despite this inability to inhibit the enzyme biochemically, tenovin 33 displayed all of the phenotypes of a DHODH inhibitor upon administration to cells. It activated p53 transcriptional activity and also brought up p53 protein levels. It also had its activation of p53 ablated upon administration of OA or uridine but was still capable of activating p53 in the presence of DHO, suggesting strongly that tenovin 33 was not targeting another enzyme, such as carbamoyl-phosphate synthetase 2, aspartate transcarbamylase, or dihydroorotase, in the *de novo* pyrimidine synthesis pathway. Whereas tenovin 39OH demonstrates that inhibition in a biochemical assay does not necessarily translate to that inhibition playing a role in the mechanism of action of the compound, tenovin 33 demonstrates that the inability of a compound to inhibit a target in a biochemical assay, or even interact with the target in solution, does not mean it is incapable of exerting an effect on that target in a cell.

Taking these observations and applying them to previously published studies utilizing the tenovins highlights the importance of considering additional targets beyond the first target found in drug discovery programs. Tenovin 6 has been previously identified as eliminating leukemic stem cells derived from patient samples (30). Now it has been established that tenovin 6 has two additional mechanisms of action, DHODH inhibition and blockage of uridine uptake, along with the previously established targets SirT1 and SirT2 (11) as well as blockage of autophagic flux (14). Thus, investigation of the effect of DHODH inhibition and nucleoside transport inhibition on leukemic stem cell populations may be of interest. This may open up the possibility for uncovering new mechanisms by which cancer stem cells may be targeted through the inhibition of DHODH or nucleoside transport (31, 32).

In conclusion, and in agreement with our previous study that demonstrates that DHODH is a remarkably frequent target (16), we show that certain tenovins are capable of inhibiting DHODH. Indeed, another frequent target, particularly of protein kinase inhibitors, is nucleoside transporters (22, 33). Certain tenovins are also capable of blocking uridine uptake into cells, which may in fact potentiate the effect of DHODH inhibition when considering the effect on tumor cell viability. One of the salient observations we made during this study is that a phenotype may be maintained throughout a compound series even while the underlying mechanism generating that particular phenotype changes. All tenovins studied here, except tenovin 50 and 50OH, are capable of activating p53 transcriptional activity, yet all display markedly different targeting profiles, as summarized in Table 2. This study also has wider implications for the drug discovery field and suggests that polypharmacology, either known or unknown, may contribute to the efficacy of many currently successful therapeutics in the clinic (34).

## Materials and methods

### Cell lines and growth conditions

ARN8 cells are derived from the parental A375 human melanoma cell line by stably transfecting the pRGCΔfos-lacZ p53-dependent reporter construct and pSV_2_neo as described previously (35). U2OS cells were purchased from ATCC (#HTB-96). HCT116 p53 WT and p53 KO cells were a kind gift from Bert Vogelstein and have been described previously (21). SKNSH p53 WT and ddp53 cells were generated previously (19). All cell culture medium was supplemented with 10% (v/v) fetal bovine serum (FBS) (Hyclone #SV30160) and 100 units ml^−1^ penicillin/streptomycin (Hyclone #SV30010) unless otherwise specified. ARN8, U2OS, and SKNSH cells were cultured in high-glucose DMEM (Hyclone #SH30243) or DMEM (Sigma–Aldrich #D6429). HCT116 p53 WT and p53 KO cells were grown in McCoy's 5A (Sigma–Aldrich #M9309) All cells were grown at 37 °C in atmospheric O_2_, 5% CO_2_, and high humidity. Passaging of cells was conducted using trypsin/EDTA (Sigma–Aldrich #T4174) detachment. All cells were counted using a Bürker cell-counting chamber. All cells were tested for mycoplasma using a commercially available kit (MycoAlert, Lonza Biosciences LT07-418).

### DHODH enzymatic assay

Enzyme assays were performed with 6 nm recombinant human DHODH prepared as described (36). The reaction mixture for these kinetic assays consisted of 1 mm DHO, 100 μm 2,3-dimethoxy-5-methyl-*p*-benzoquinone (Sigma–Aldrich #D9150), and 100 μm 2,6-dichlorophenolindophenol sodium salt (DCIP; Sigma–Aldrich #D1878) in enzyme buffer (50 mm Tris-HCl pH 8.0, 0.1% Triton X-100, 150 mm KCl). A stock solution of 20 mm DCIP was prepared in enzyme buffer and filtered through filter paper (20−25-μm pore size) just before use. Loss in absorbance by DCIP was measured at 595 nm at room temperature in a stepped time course (8 × 2 min, 8 × 3 min, 6 × 5 min). The observed decrease in absorbance over time was linear between 8 and 26 min. Therefore, for each concentration of inhibitor tested, a value for DHODH's *V*_max_ was estimated by linear regression within this time frame. The IC_50_ is defined as the concentration of inhibitor that gives *V*_max_([I]) = *V*_max_(DMSO)/2.

### Thermal shift assay

5 μm recombinant human DHODH prepared as described (36) was used in this assay. The assay relied upon the intrinsic fluorescence of the embedded flavin mononucleotide (FMN) within the DHODH as a proxy for unfolding of DHODH across a temperature range, much in the same manner as the thermal shift assay using SYPRO Orange (37). A PCR machine with a filter set with an excitation range of 475–500 nm and an emission range of 520–590 nm was used for this experiment. 5 μm DHODH was prepared with 200 μm compound with a final concentration of 0.5% DMSO in each sample in buffer consisting of 50 mm HEPES and 150 mm NaCl. The final assay volume was 10 μl. The samples were heated across a temperature range with the fluorescence emission from FMN monitored at each temperature.

### Co-crystallization of tenovin 6 and human DHODH

Co-crystals were prepared as described (36). In short, 18 mg ml^−1^ DHODH (dissolved in 10 mm *N*,*N*-dimethylundecylamine *N*-oxide (C11DAO, Fluka), 400 mm NaCl, 1 mm EDTA, 100 mm HEPES (pH 7), and 30% glycerol) was mixed in a 1:1 ratio with 1.82 m NH_4_SO_4_, 0.1 m sodium acetate, pH 4.8, 40 mm *N*,*N*-dimethyldodecylamine *N*-oxide, 6.4 mm C11DAO, 2 mm l-dihydroorotic acid, and 1 mm tenovin 6. The crystals did grow from hanging drops at 20 °C in a VDX plate: 2.5 μl of protein solution mixed with 2.5 μl of reservoir (30% (v/v) glycerol, 0.1 m sodium acetate, pH 4.8, and 1.8 m NH_4_SO_4_). A crystal was flash-frozen in liquid nitrogen, and data were collected to 1.85 Å at MAX IV laboratory, beamline I911-3. The structure was determined using molecular replacement and refined in Refmac5 (38). The coordinates and the structure factors have been deposited in the Protein Data Bank (PDB code 6GK0). Details of the crystal structure parameters can be found in Table S1.

### IncuCyte cell confluence measurements

ARN8 or HCT116 p53 WT or KO cells were seeded in a 96-well plate at a density of 4000 cells/well in a volume of 100 μl. The cells were incubated as described above for about 24 h prior to treatment. The medium of the assigned wells was replaced with 100 μl of fresh medium containing a 5 μm concentration of the corresponding Tenovins or DMSO control and placed in an IncuCyte S3 system located inside a CO_2_ incubator. The cell growth was monitored every 2 h for a total of 72 h after an initial 30-min incubation period. The cell confluence percentage was analyzed by IncuCyte S3 2019 Rev 1 software (Essen Biosciences).

### SRB assay

Cells were seeded in a 96-well plate at a density of 500 cells/well (ARN8) in a volume of 100 μl of fully supplemented growth medium and incubated as described above for 24 h. SKNSH p53 WT pCMV neo cells were seeded at 7500 cells/well in 100 μl of fully supplemented growth medium and incubated as described above for 24 h. SKNSH ddp53 cells were seeded at a density of 6000 cells/well in 100 μl of fully supplemented growth medium and incubated as described above for 24 h. Cells were checked for adherence and treated with tenovins in DMSO supplemented with 100 μm uridine, 1 mm DHO, or 1 mm OA. The final percentage of DMSO per well was <0.2%. Cells were incubated for 72 h with compounds. Following incubation, medium was removed from the cells and replaced by 150 μl of 1× sterile PBS and 50 μl of 40% (w/v) TCA (Sigma–Aldrich #T9159) in distilled H_2_O and incubated at 4 °C for 1 h to fix the cells. The plate was then washed with three changes of water with all liquid allowed to drain from the plate. 50 μl of 0.4% (w/v) sulforhodamine B (Sigma–Aldrich #230162) with 1% (v/v) acetic acid (Sigma–Aldrich #320099) in H_2_O was added to each well and incubated for 30 min. Excess dye was washed out with three changes of 1% (v/v) acetic acid in H_2_O. The remaining dye was solubilized in 100 μl of 10 mm unbuffered Tris-base (Sigma–Aldrich #93362) per well, and absorbance was read at 490 nm on a spectrophotometer.

### p53 transcriptional activation (CPRG) cell reporter assay

ARN8 cells were seeded in a 96-well plate at a density of 20,000 cells/well in a volume of 100 μl of fully supplemented growth medium. Following a 24-h incubation, the cells were treated with 100 μl of the corresponding compound titration diluted in growth medium either nonsupplemented or supplemented with 100 μm uridine, 1 mm DHO, or 1 mm OA. After 18 h of treatment, the medium was removed, and the cells were washed once with 1× PBS (Hyclone). Subsequently, 50 μl of 1× reporter lysis buffer (Promega #E4030) was added to each well, and the plates were stored at –20 °C for at least 2 h. After thawing the cell lysate, 150 μl of CPRG mix consisting of 0.1 m phosphate buffer, pH 7.5 (0.2 m Na_2_HPO_4_, 0.2 m NaH_2_PO_4_, distilled H_2_O), 4 mg ml^−1^ chlorophenol red-β-d-galactopyranoside monosodium salt (Roche Applied Science #884308) diluted in 0.1 m phosphate buffer, pH 7.5, 0.1 m MgCl, 4.5 m β-mercaptoethanol (Sigma–Aldrich #M6250) was added to each well. β-Gal activity was measured after 24 h at 590 nm on a spectrophotometer.

### Clonogenic assay

ARN8 cells were seeded at 10,000 cells/well in 12-well plates and incubated for 24 h. All compounds were diluted to 10× stock in fully supplemented growth medium prior to addition to wells. Cells were grown for 72 h in the presence of the compounds and supplemented as indicated with either 100 μm uridine, 1 mm DHO, or 1 mm OA. Following treatment, medium was removed, and cells were washed twice with fully supplemented growth medium and then grown in fully supplemented medium for 24 h prior to fixation unless otherwise stated. Following the recovery phase, growth medium was removed, and each well was washed twice with 1× PBS. Cells were fixed using 50:50 methanol/acetone (v/v) and incubated at –20 °C for 10 min. The solvents were removed, and cells were left to air-dry. Cells were stained with Giemsa stain (Sigma–Aldrich #48900) diluted to 7.5% (v/v) in 1× PBS. Cells were then washed with warm water to remove excess stain and allowed to dry.

### Flow cytometry

ARN8 cells were seeded at 30,000 per well, respectively, in 6-well plates and incubated for 24 h. All compounds were diluted to 10× stocks in fully supplemented growth medium. Cells were incubated with the compounds for 48 h. Cell culture medium was removed and placed into Falcon tubes. Wells were washed twice with 1× PBS with the washes saved in the Falcon tubes to harvest floating dead cells. Cells remaining in the wells were trypsinized with 200 μl of 1× trypsin/EDTA (Sigma–Aldrich #T4174). Following detachment, fresh growth medium was added to each well, and the contents were removed and placed in the relevant tubes. Any remaining cells in the plate were then gathered by washing twice with 2 ml of 1× PBS with the washes saved and added to the relevant tubes. The tubes were centrifuged at 500 × *g* for 5 min. Cell pellets were washed twice with 1× PBS. The pellets were resuspended in 1 ml of 1× PBS and added dropwise to 3 ml of 99.9% ethanol while vortex mixing at high speed. Cells were then fixed at –20 °C for 24 h. Following fixation, cells were pelleted at 1390 × *g* for 5 min. The cells were washed three times with 2 ml of 1× PBS containing 3% FBS with centrifugation at 1390 × *g* between washes. Cells were resuspended in 0.5 ml of PBS containing 10 μg of propidium iodide (Invitrogen #P3566) and 50 μg of RNase A (Sigma–Aldrich #R4642) and incubated on ice in the dark for 10 min. Flow cytometry was performed using a BD Biosciences FACScan or FACSCalibur with 10,000 events counted within the gating region.

### Western blotting

150,000 ARN8, HCT116 p53 WT, or HCT116 p53 KO cells were seeded on 6-well plates in 2 ml of fully supplemented medium as described above. Following 24 h of incubation, cells were treated with compounds for the indicated amount of time in the presence of either no supplement, 100 μm uridine, 1 mm DHO, or 1 mm OA. Cells were harvested using 150 μl of 1× Bio-Rad Laemmli loading buffer without bromphenol blue (Bio-Rad #1610747). A protein assay was conducted using the Bio-Rad DC protein analysis kit (Bio-Rad #5000111), and the protein levels were normalized between samples. DTT (Sigma–Aldrich #43819) to a final concentration of 100 μm was added to each sample. The ladder used on blots is the Bio-Rad All Blue (Bio-Rad #1610373). The samples were run on 4–15% 12-well stain-free TGX gels (Bio-Rad #4568085) in standard Tris-glycine running buffer (Bio-Rad #1610732) at 150 V. Prior to transfer, the gels were activated using the ChemiDoc Touch (Bio-Rad #1708370) stain-free gel activation protocol for 5 min. The gel was then blotted using the Trans-Blot Turbo transfer system (Bio-Rad #1704150) using the polyvinylidene difluoride membrane Trans-Blot Turbo kit (Bio-Rad #1704272) for sandwich assembly. The transfer was conducted using the Trans-Blot Turbo preset program for standard semidry transfer (30 min). All membranes were blocked in 5% milk (w/v) in PBS-T containing 0.1% Tween 20 (v/v) (Sigma–Aldrich P9416). All antibodies were made up in 5% milk (w/v) in PBS-T. All antibody incubations were either overnight at 4 °C or at room temperature for 1 h. Primary antibodies for p53 were mouse monoclonal DO1 (Abcam #1101) diluted to 1:1000 (v/v), GAPDH mouse monoclonal 6C5 (Abcam #8245), ENT1 (Abcam #ab135756), ENT2 (Sigma–Aldrich #SAB1405951), p21/WAF1/CIP1 (Calbiochem #OP64), and HDM2 (Calbiochem #OP46). All secondary antibodies were polyclonal rabbit anti-mouse antibodies conjugated to horseradish peroxidase (DAKO, Glostrup, Denmark #P0260) and diluted at concentrations of 1:2000 (v/v). Imaging of the blots used the ChemiDoc Touch system for both the chemiluminescence mode for antibody detection and development using the Clarity substrate (Bio-Rad #1705060). All blots were normalized to their respective vehicle control and quantified using ImageLab where quantification was required (Bio-Rad version 5.2.1 build 11).

### Uridine uptake assay

100,000 (24-h assay) or 200,000 (15-min assay) U2OS or ARN8 cells were seeded per well in 6-well plates in 2 ml of fully supplemented culture medium as described above. 24 h postseeding, medium was removed, and cells were treated with the indicated compounds for either 15 min or 24 h in 2 ml of FBS-free medium supplemented with serum replacement 3 (Sigma–Aldrich #S2640). At the end of the treatment, an extra well of each treatment was harvested in 100 μl of 1× LDS (62.5 mm Tris-HCl, pH 6.8, 10% glycerol, 1% LDS) for quantification of protein levels to determine the level of cell death after 24 h for normalization with protein concentration determined by the Bio-Rad DC protein assay (Bio-Rad #5000111). [^3^H]Uridine (PerkinElmer Life Sciences #NET367001MC) was prepared in serum replacement medium to a final emission of 3.65 μCi ml^−1^ and added to cells for 10 min. Following 10 min, the cells were washed with four quick changes of 1 mm unlabeled uridine prepared in ice-cold transport buffer (20 mm Tris-HCl, pH 7.4, 130 mm NaCl, 3 mm K_2_HPO_4_, 1 mm MgCl_2_, 5 mm glucose, 2 mm CaCl_2_). The samples were then harvested in 100 μl of 10% SDS. 900 μl of Optiphase Supermix (PerkinElmer Life Sciences #1200-439) was added to each sample, and the cpm were measured using a 1450 MicroBeta JET for liquid scintillation (PerkinElmer Life Sciences/Wallac).

### MD simulations

##### System preparation

The Protein Data bank contains 18 crystal structures of the enzyme DHODH (human) and 11 crystal structures of DHODH from nonhuman sources. We chose the structure with PDB ID 2WV8 (39) for our modeling studies as it had complete coordinates for the longest resolved sequence (from residue number 32 to 396), resolved at 1.9 Å. This structure was crystallized in complex with a small molecule, which was removed for our docking studies.

##### Ligand preparation

The three-dimensional structures of the tenovins were built using *Maestro* and minimized using the *Macromodel* module employing the OPLS-2005 force field (40) in Schrodinger 9.0. All of the inhibitors were then prepared with *Ligprep*, which generates low energy tautomers and enumerates realistic protonation states of the inhibitors at physiological pH.

##### Ligand docking

The prepared inhibitors were docked into the binding pockets of the chosen structure of DHODH using *Glide* (41). A box of size 10 × 10 × 10 Å for molecular docking centered on the selected active site residues (the active site was defined as the region where all of the small molecules were bound in the various co-crystal structures) was used to restrict the search space of each docked ligand. Default *Glide* settings were used to generate the grids. A rigid receptor and flexible ligands were used during the docking. The docked conformation of each ligand was evaluated using the *Glide* Extra Precision (XP) scoring function. The effects of conformational flexibility of the protein were incorporated by carrying out docking on conformational substates of the DHODH enzyme generated using MD simulations (see below). The top scoring binding pose was selected for further refinement.

### MD simulations

The apo-DHODH and the modeled DHODH-tenovin complexes were subject to refinement using MD simulations. The simulations were carried out using the *pemed.CUDA* module of the program Amber14 (42). The partial charges and force field parameters for the cofactors and the tenovins were generated using the *Antechamber* module in Amber. All atom versions of the Amber 14SB force field (ff14SB) (43) and the general Amber force field (44) were used for the protein and the cofactors/tenovins, respectively. The *Xleap* module was used to prepare the system for the MD simulations. Each simulation system was neutralized with an appropriate number of counterions. Each neutralized system was solvated in an octahedral box with TIP3P (45) water molecules, with at least a 10-Å boundary between the solute atoms and the borders of the box. During the simulations, LJ and short-range electrostatic interactions were treated with a cut-off scheme, and the long-range electrostatic interactions were treated with the particle mesh Ewald method (46) using a real space cut-off distance of 9 Å. The Settle (47) algorithm was used to constrain bond vibrations involving hydrogen atoms, which allowed a time step of 2 fs during the simulations.

Solvent molecules and counterions were initially relaxed using energy minimization with restraints on the protein and inhibitor atoms. This was followed by unrestrained energy minimization to remove any steric clashes. Subsequently, the system was gradually heated from 0 to 300 K using MD simulations with positional restraints (force constant: 50 kcal mol^−1^ Å^−2^) on protein and inhibitors over a period of 0.25 ns, allowing water molecules and ions to move freely. During an additional 0.25 ns, the positional restraints were gradually reduced, followed by a 2-ns unrestrained MD simulation to equilibrate all of the atoms. For each system, a 250-ns production MD run at 300 K in the NPT ensemble was conducted in triplicate (assigning different initial velocities to propagate each MD simulation). Simulation trajectories were visualized using VMD (48), and figures were generated using PyMOL (49).

### Binding energy calculations

The binding free energies between the tenovins and DHODH were calculated using the MMPBSA (molecular mechanics Poisson–Boltzmann surface area) methodology (50, 51, 52). 500 conformations from the last 50 ns of the MD simulations of each DHODH-tenovin complex were taken, and water molecules, counterions, and membrane atoms were removed. Binding free energies (Δ*G*_bind_) were calculated for each conformation using the following,

(Eq. 1) $$\Delta G_{\text{bind}}=G_{\text{complex}}-\left(G_{\text{receptor}}+G_{\text{ligand}}\right)$$

where

(Eq. 2) $$\Delta G_{\text{bind}}=\Delta G_{\text{MM}}+\Delta G_{\text{sol}}-T\Delta S$$

Δ*G*_MM_ is the change in the molecular mechanics energy upon complexation in the gas phase, Δ*G*_sol_ is the change in solvation free energy, and *T*Δ*S* is the change of conformational entropy associated with ligand binding. The molecular mechanics free energy (Δ*G*_MM_) is further split into Van der Waals (Δ*G*_vdw_) and electrostatic (Δ*G*_ele_) energies.

(Eq. 3) $$\Delta G_{\text{MM}}=\Delta G_{\text{ele}}+\Delta G_{\text{vdw}}$$

The solvation free energy Δ*G*_sol_ arises from polar (electrostatic) solvation free energy (Δ*G*_PB_) and nonpolar solvation free energy (Δ*G*_SA_) as in Equation 4,

(Eq. 4) $$\Delta G_{\text{sol}}=\Delta G_{\text{PB}}+\Delta G_{\text{SA}}$$

ΔG_PB_ is computed by solving the linearized Poisson–Boltzmann equation using Parse radii and a solvent probe radius of 1.4 Å. In these calculations, the dielectric constant was set to 1.0 for the interior of the solutes and 80.0 for the solvent. Δ*G*_SA_ was determined using a solvent-accessible surface area (SASA)–dependent term as in Equation 5,

(Eq. 5) $$\Delta G_{\text{SA}}=\gamma \times \text{SASA}+\beta$$

where γ is the surface tension proportionality constant and was set to 0.00542 kcal mol^−1^·Å^−2^, and β is the offset value, which was 0.92 kcal mol^−1^ here.

Relative binding free energies were calculated using alchemical transformation methods (thermodynamic integration (TI) and free energy perturbation (FEP)) discussed in our previous study (53). These advanced methods used here were motivated by several recent studies where TI- and FEP-based calculations were shown to reproduce experimentally determined mutation-dependent binding free energy differences with root mean square errors of ∼1 kcal/mol (54, 55, 56, 57, 58). In our study, the free energy differences between two ligands were calculated by gradually perturbing the structure of one ligand (Lig1) to the other ligand (Lig2) in a series of discrete steps, represented by λ values. The λ values vary from 0 to 1 corresponding, respectively, to the two structures. Two different transformations needed to be simulated: Lig1 to Lig2 in their apo-states and Lig1 to Lig2 complexed with DHODH bound to the model membrane. We used a total of 11 λ windows (0.0, 0.10, 0.20, 0.30, 0.40, 0.50, 0.60, 0.70, 0.80, 0.90, 1.0) for the TI and 19 λ windows (0.05, 0.10, 0.15, 0.20, 0.25, 0.30, 0.35, 0.40, 0.45, 0.50, 0.55, 0.60, 0.65, 0.70, 0.75, 0.80, 0.85, 0.90, 0.95) for the FEP/multi-Bennett acceptance ratio (MBAR) simulations at 300 K. At each λ window, MD simulations were carried out for 5 ns. Free energy derivatives (∂*V*/∂λ) were collected independently for each λ from the production run. In the TI method, the free energy difference is calculated from the integral of ∂*V*(λ)/∂λ from 0 to 1, where *V* is the potential energy. In the case of FEP, free energy differences were calculated using the MBAR method as implemented in Amber 16. The transformations were always between Tenovin 6 and each of the other six ligands, and hence the binding free energies of each of the six ligands relative to that of tenovin 6 are computed.

### Per-residue decomposition

DHODH residues that contribute the most to the interactions with the tenovins were identified by decomposing the binding energies into contributions from individual residues; this was carried out using the MMGBSA energy decomposition scheme. The MMGBSA calculations were carried out in the same way as in the MMPBSA calculations. The polar contribution to the solvation free energy was determined by applying the generalized born method (igb = 2) (42), using mbondi2 radii for the atoms. The nonpolar contributions were estimated using the ICOSA method (42) by computing a SASA-dependent term using a surface tension proportionality constant of 0.0072 kcal mol^−1^ A^2^.

### Adaptive steered molecular dynamics (ASMD)

Ligand-pulling simulations were carried out using ASMD method (59). In the ASMD method, the overall reaction coordinate is divided into segments, and the potential of mean force (PMF) is calculated over each segment within an SMD-like stage using the Jarzynski equality. All ASMD simulations were carried out with protocols similar to those mentioned above but with increased size of the waterbox. Each DHODH–ligand complex system was solvated in an octahedral box with TIP3P (45) water molecules, with at least a 15-Å boundary between the solute atoms and the borders of the box. In the current work, the distance between the center of the mass of the ligand binding site residues (heavy atoms) and center of mass of the ligand heavy atoms was used as a reaction coordinate, and the ligand was slowly pulled out in three segments with distances from 3 to 12 Å. 25 simulations were carried out for each segment with pulling speed of 10 Å ns^−1^ and a force constant of 7.2 kcal mol^−1^. At the end of each stage, PMF was calculated to identify the simulations where the work done is closest to the Jarzynski average, and coordinates and velocities were selected for all trajectories in the subsequent stage. Finally, the overall PMF was constructed using the PMF constructed from each stage. PMF was calculated with the Python scripts available from the ASMD developers.

## Data availability

All data described are included within the article. All raw data from figures contained within this article and the supporting data have been published online at Mendeley and can be accessed by the following DOI: 10.17632/k77vyybbr4.1. For the co-crystal structure of tenovin 6 and DHODH, the coordinates and the structure factors have been deposited in the Protein Data Bank (PDB code 6GK0). Replicates of representative experiments shown in the main figures can be requested from the corresponding authors, Marcus Ladds (m.ladds@beatson.gla.ac.uk) and Sonia Laín (sonia.lain@ki.se).
