# Supplementary material for: Exploitation of dihydroorotate dehydrogenase (DHODH) and p53 activation as therapeutic targets: A case study in polypharmacology
Source: J Biol Chem. 2021 Jan 13;295(52):17935–49. doi: 10.1074/jbc.RA119.012056 (PMC7939445; doi:10.1074/jbc.RA119.012056)
Supplement: Supplementary file 1 [file mmc1.pdf]

Exploitation of DHODH and p53 activation as therapeutic targets - a case study in polypharmacology (SUPPLEMENTAL DATA)

Marcus J G W Ladds[1,2*#], Gergana Popova[1@], Andrés Pastor-Fernández[1,2@], Srinivasaraghavan Kannan[3], Ingeborg M M van Leeuwen[1], Maria Håkansson[4], Björn Walse[4], Fredrik Tholander[5], Ravi Bhatia[6], Chandra S. Verma[3,7,8], David P Lane[1,2], and Sonia Laín[1,2*]

From the [1] Department of Microbiology, Tumor and Cell Biology, Karolinska Institutet, Stockholm SE 171-77, Sweden; [2] SciLifeLab, Department of Microbiology, Tumor and Cell Biology, Karolinska Institutet, Tomtebodavägen 23, SE-171 21, Sweden; [3] Bioinformatics Institute (BII), A*STAR, 30 Biopolis Street, 07-01 Matrix, 138671, Singapore; [4] SARomics Biostructures AB, Medicon Village, SE-223 81, Lund, Sweden; [5]Department of Medical Biochemistry and Biophysics, Tomtebodavägen 23, SE-171 21, Sweden; [6] Division of Hematology & Oncology, Comprehensive Cancer Center, 1720 2nd Avenue South, NP2540, Birmingham, AL 35294-3300, USA; [7] Department of Biological Sciences, National University of Singapore, 16 Science Drive, Singapore 117558; [8] School of Biological Sciences, Nanyang Technological University, 60 Nanyang Drive, Singapore 637551

Running title: *Exploitation of DHODH and p53 as therapeutic targets*

@ These authors contributed equally.

# Present Address: Cancer Research UK Beatson Institute, Switchback Rd, Glasgow G61 2BD, UK.

* To whom correspondence should be addressed: Marcus J.G.W Ladds: Cancer Research UK Beatson Institute, Switchback Rd, Glasgow, G61 2BD, UK m.ladds@beatson.gla.ac.uk; Tel. +44 141 330 6591. Sonia Laín: Department of Microbiology, Tumor and Cell Biology, Biomedicum, Solnavägen 9, 171 65 Solna, Stockholm, Sweden, sonia.lain@ki.se; Tel. +46 72 155 08 77

Supplemental Figure 1

A

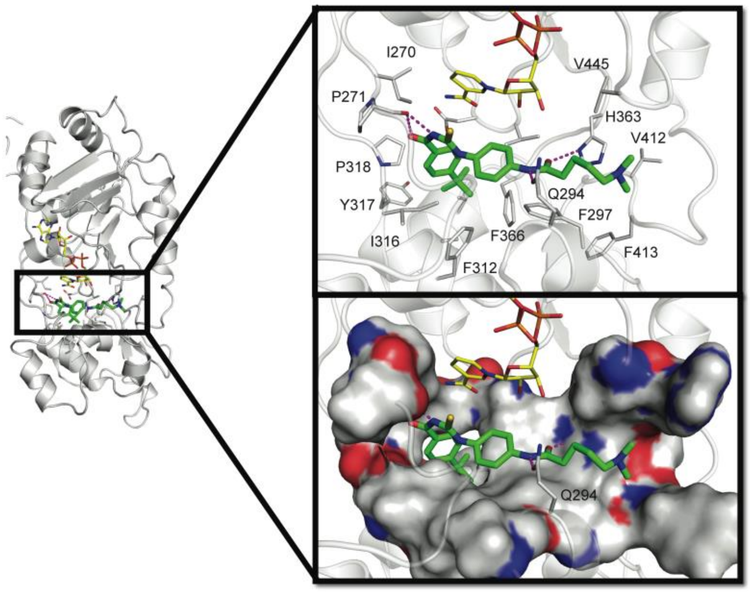

B

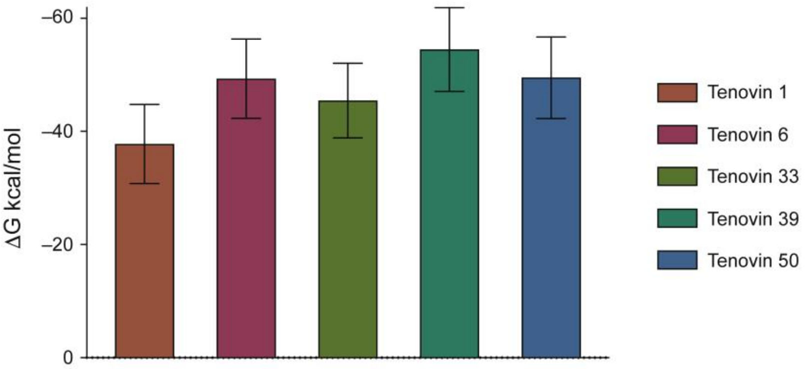

C

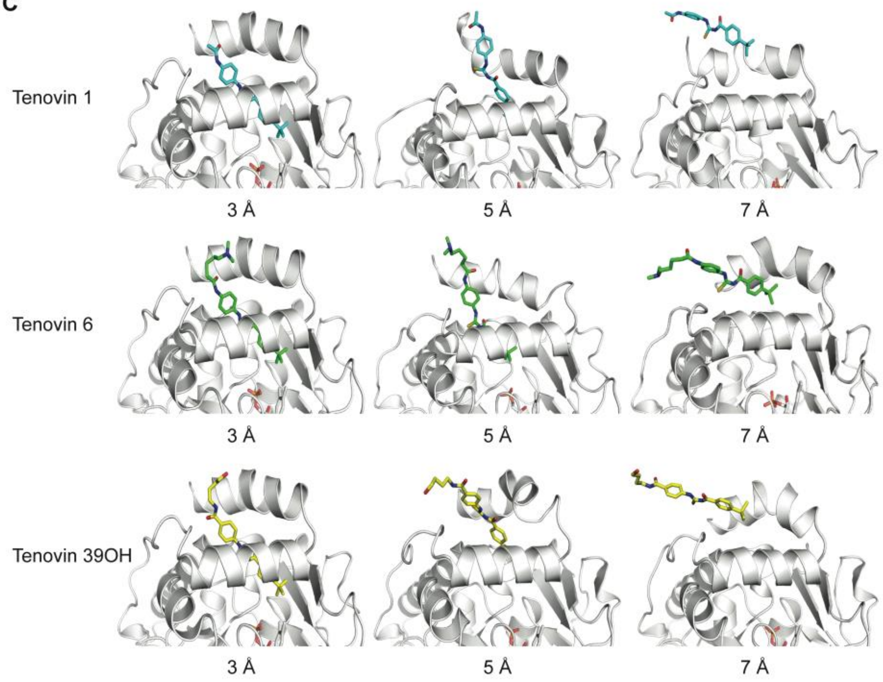

Supplemental Figure 1

(A) Predicted binding mode of tenovin 6 with the enzyme Sirtuin1 (SirT1). Sirtuin1 is shown as cartoon (grey colour) and the cofactor NAD (yellow carbon) and bound tenovin 6 (green carbon) are shown as thick lines. The tenovin binding pocket residues are shown as thin lines with the hydrogen bond interactions between SirT1 and the tenovin shown as dashed lines (magenta). (B) Binding free energies between SirT1 and the tenovins calculated using the MMPBSA approach averaged over the conformations sampled during the MD simulations. The error bars (standard deviation) are indicated. (C) The position of either tenovins 1, 6 or 39OH at each unit of distance (Å) in the pulled ligand simulation that has been graphed in figure 1E.

Supplemental Figure 2

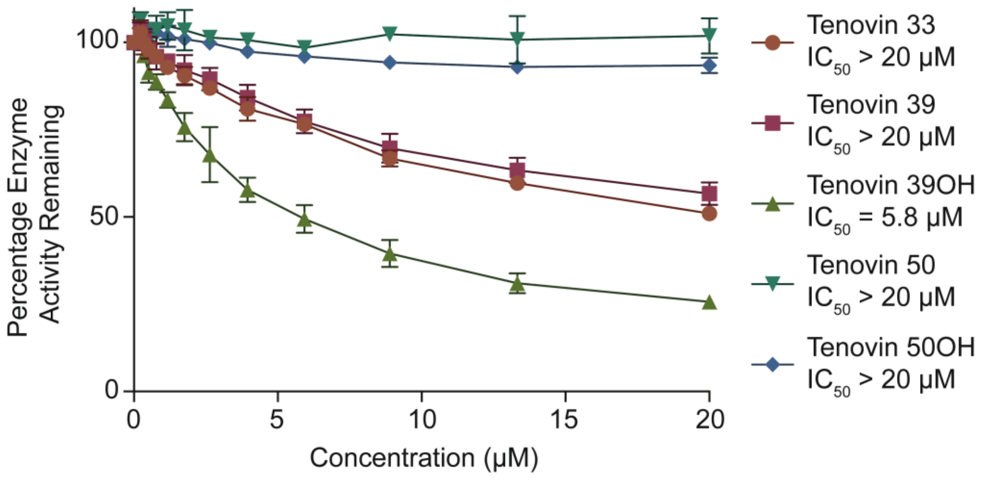

Supplemental Figure 2

Values obtained using a kinetic DHODH enzyme assay for each tenovin indicated. Values correspond to the average of 3 independent repeats ± SD with 3 technical repeats each.

Supplemental Figure 3

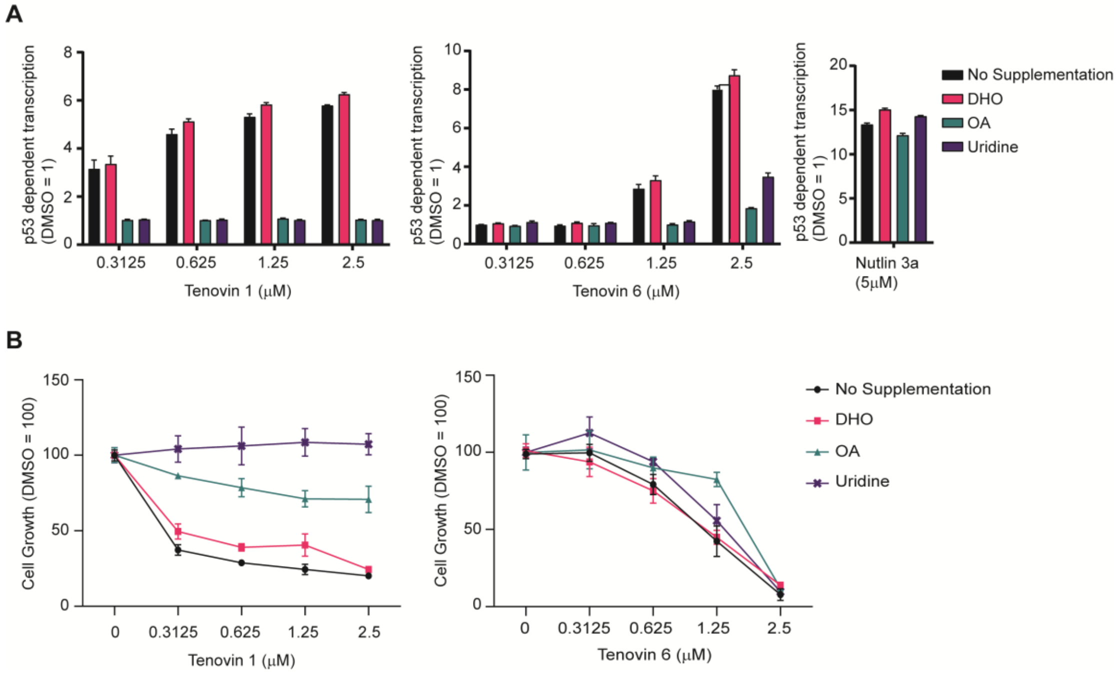

Supplemental Figure 3

(A) p53 transcriptional activity assay (CPRG) in ARN8 cells treated with a low dose of tenovin 1, tenovin 6 or 5 μM nutlin 3a as a positive control and either supplemented with vehicle, DHO, OA or uridine. This panel supplements figure 4a. (B) SRB assay in ARN8 cells treated with low dose tenovin 1 or tenovin 6 for 72 h supplemented with DHO, OA or uridine. This panel supplements figure 4c.

Supplemental Figure 4

Figure 3a whole blots

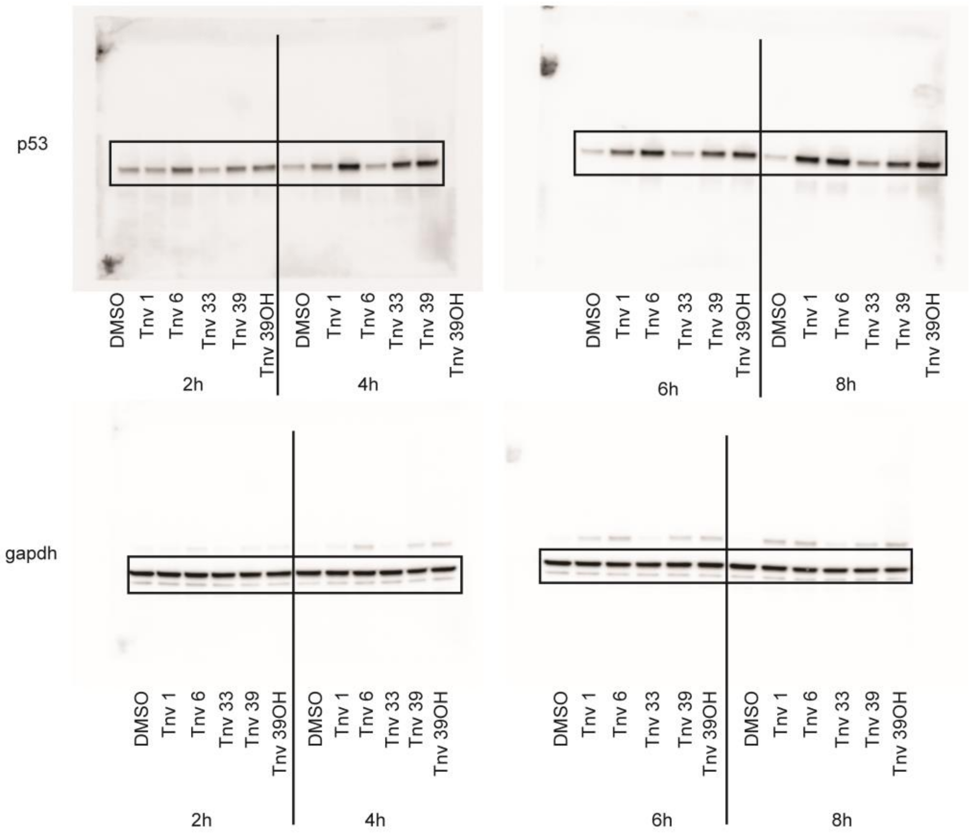

Figure 3d whole blots

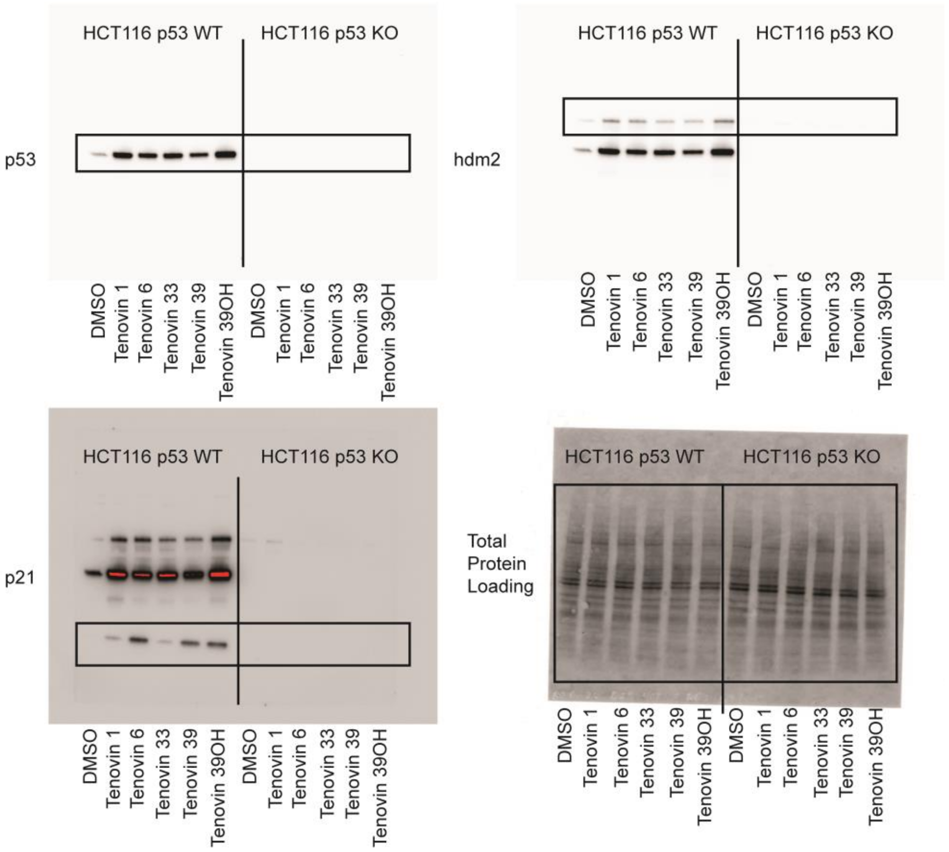

Supplemental Figure 4 continued

Figure 4b whole blots

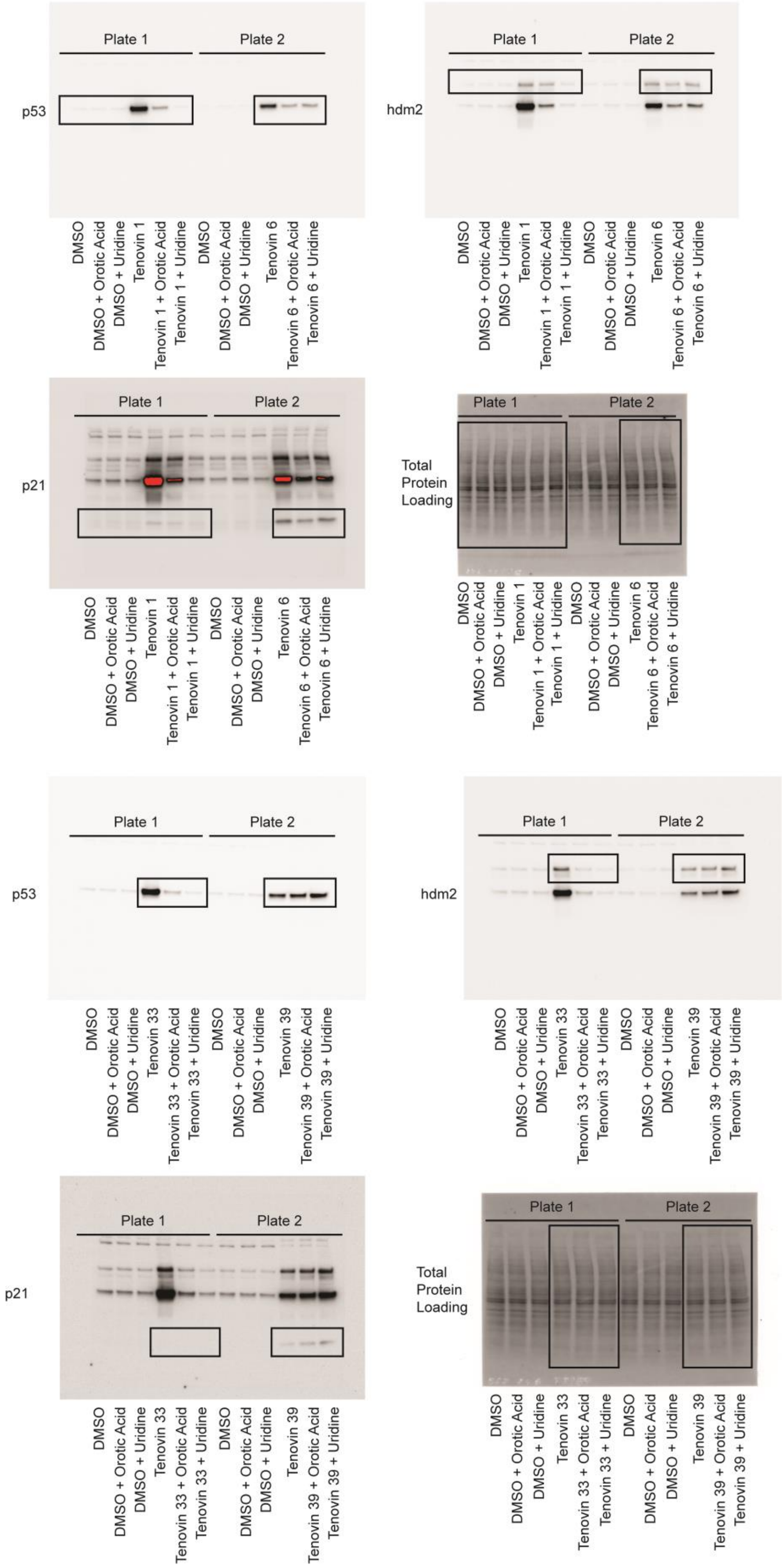

Supplemental Figure 4 continued

Figure 4b whole blots

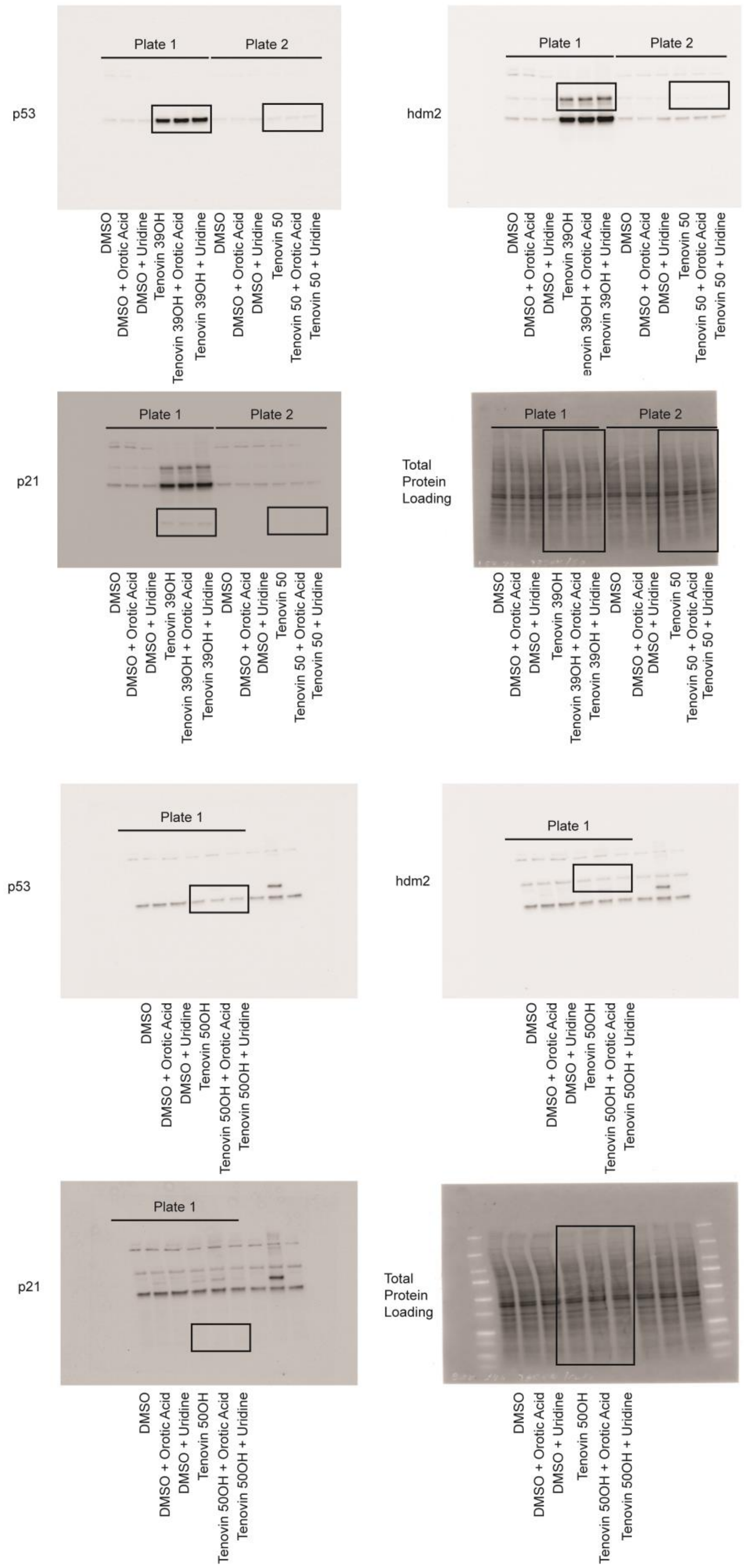

Supplemental Figure 4

Figure 4b whole blots continued

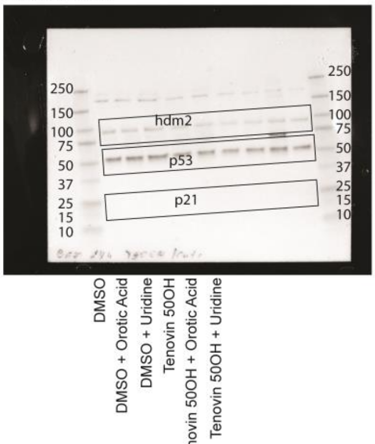

Figure 6b whole blots

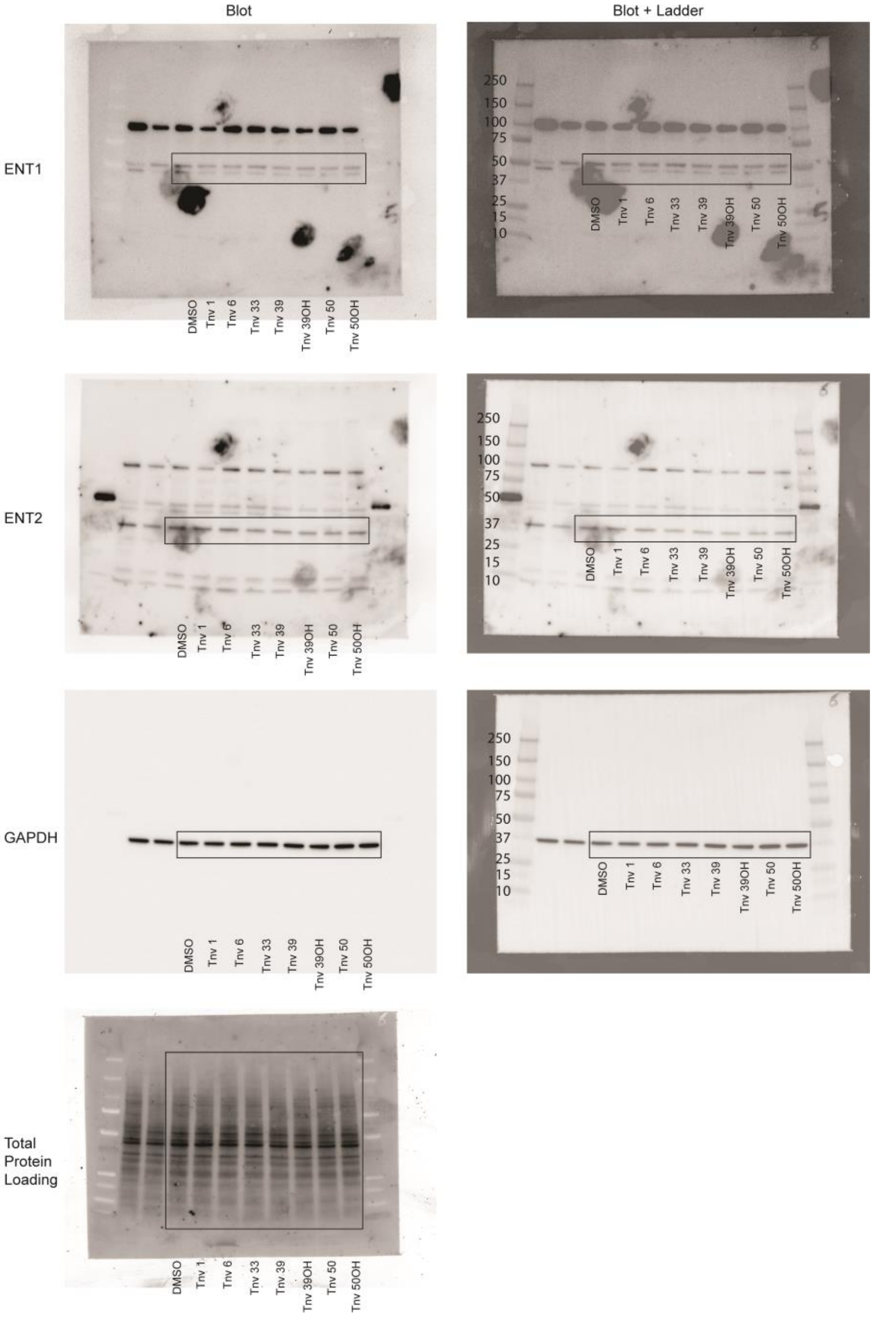

Supplemental Figure 4

Total blot images for figures 3a, 4b and 6b. Areas highlighted are the regions of the blot that have been used in the figures. Antibodies were used sequentially on each blot without stripping the blot between primary antibodies. For figure 4b blots, each blot has its own DMSO control from the same treatment plate next to it, though for clarity only one control was shown on the figure. The total protein loading controls have been shown here in full. The total protein loading controls have been processed as per manufacturer's instructions (1).

Table S1: Summary of structural determination of DHODH in complex with tenovin 6.

**Ligands**	FMN, L-DHO, Tenovin, DDQ, glycerol, sulphate, acetic acid, chloride
**Resolution (Å)**	20-1.85 (1.89-1.85)
**Wavelength (Å)**	1,0000
**Space Group**	$P3_221$
**Unit Cell (Å)**	A=90.5, b=90.5, c=122.8
**Completeness (%)**	99.8 (99.5)
**Number of observations/unique reflections**	223 553 / 50 141
**<I/σ(I)>**	5.4 (1.2)
**CC(1/2) (%)**	97.9 (41.3)
**R_{merge} (I) (%)**	17.6 (105.8)
**R_{cryst} (F) (%)**	16.5
**R_{free} (F) (%)**	19.7
**No. of non-hydrogen atoms**	3209
**No. of water molecules**	228
**Rms deviations from ideal geometry: Bond lengths (Å)**	0.022
**Bond angles (deg)**	2.1
**Mean B-factor protein (Å^2)**	26.0
**Mean B-factor (solvent, Å^2)**	32.9
**Mean B-factor (ligand tenovin, Å^2)**	53.9
**Mean B-factor (other ligands, Å^2)**	37.2

Supplementary References

1. Gurtler A, Kunz N, Gomolka M, Hornhardt S, Friedl AA, McDonald K, et al. Stain-Free technology as a normalization tool in Western blot analysis. Anal Biochem. 2013;433(2):105-11.
